# Inhibition of vascular smooth muscle cell PERK/ATF4 ER stress signaling protects against abdominal aortic aneurysms

**DOI:** 10.1172/jci.insight.183959

**Published:** 2025-01-23

**Authors:** Brennan Callow, Xiaobing He, Nicholas Juriga, Kevin D. Mangum, Amrita Joshi, Xianying Xing, Andrea Obi, Abhijnan Chattopadhyay, Dianna M. Milewicz, Mary X. O’Riordan, Johann Gudjonsson, Katherine Gallagher, Frank M. Davis

**Affiliations:** 1 Section of Vascular Surgery, Department of Surgery, and; 2 Department of Dermatology, University of Michigan, Ann Arbor, Michigan, USA.; 3 University of Texas Health Science Center at Houston, Houston, Texas, USA.; 4 Department Microbiology and Immunology, University of Michigan, Ann Arbor, Michigan, USA.

**Keywords:** Vascular biology, Cardiovascular disease, Cell stress, Surgery

## Abstract

Abdominal aortic aneurysms (AAA) are a life-threatening cardiovascular disease for which there is a lack of effective therapy preventing aortic rupture. During AAA formation, pathological vascular remodeling is driven by vascular smooth muscle cell (VSMC) dysfunction and apoptosis, for which the mechanisms regulating loss of VSMCs within the aortic wall remain poorly defined. Using single-cell RNA-Seq of human AAA tissues, we identified increased activation of the endoplasmic reticulum stress response pathway, PERK/eIF2α/ATF4, in aortic VSMCs resulting in upregulation of an apoptotic cellular response. Mechanistically, we reported that aberrant TNF-α activity within the aortic wall induces VSMC ATF4 activation through the PERK endoplasmic reticulum stress response, resulting in progressive apoptosis. In vivo targeted inhibition of the PERK pathway, with VSMC-specific genetic depletion (*Eif2ak3^fl/fl^ Myh11-CreER^T2^*) or pharmacological inhibition in the elastase and angiotensin II–induced AAA model preserved VSMC function, decreased elastin fragmentation, attenuated VSMC apoptosis, and markedly reduced AAA expansion. Together, our findings suggest that cell-specific pharmacologic therapy targeting the PERK/eIF2α/ATF4 pathway in VSMCs may be an effective intervention to prevent AAA expansion.

## Abstract 2

Pathological activation of the PERK/ATF4 ER stress response drives vascular smooth muscle cell dysfunction during abdominal aortic aneurysm formation and represents a therapeutic target.

## Graphical abstract

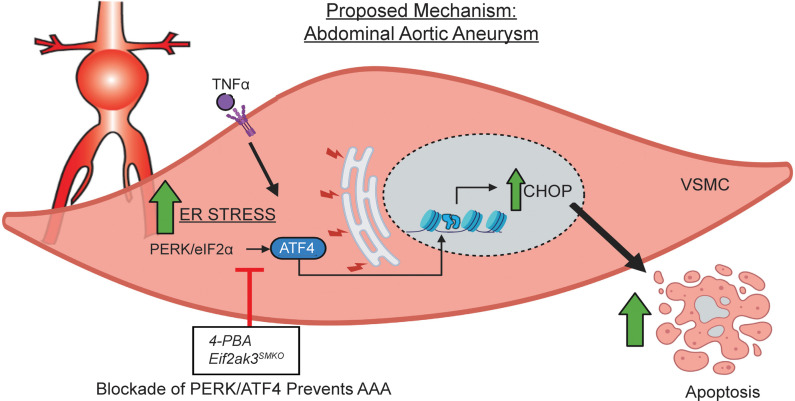

## Introduction

Abdominal aortic aneurysms (AAAs) are a common vascular disease, present in greater than 1 million people in the United States, that can progress to the potentially fatal consequence of aortic rupture, which carries a mortality rate of approximately 90% (1–3). AAAs usually affect the infrarenal region of the aorta and have associated risk factors, including male sex, advanced age, smoking, and atherosclerosis (4). Currently there are no prove pharmacological therapies that can slow the growth of AAAs or prevent aortic rupture; therefore, operative repair represents the only treatment with significant morbidity (5). Within the aorta, vascular smooth muscle cells (VSMCs) are critical to maintaining the integrity of the vessel wall and retain remarkable plasticity in response to environmental stimuli. Prior investigations into the mechanisms that drive AAA formation have consistently highlighted VSMC loss through apoptosis as pathological hallmarks of AAA development (6). Within patient clinical samples and animal models, loss of VSMCs within the aortic wall plays a critical role in AAA development and potential rupture (7–10). Identifying the mechanisms that drive VSMC dysfunction and apoptosis during aortic dilation is a crucial step toward developing effective therapeutics.

The endoplasmic reticulum (ER) is a major signal-transducing organelle that senses and responds to changes in cellular homeostasis. Specifically, disturbances in the structure and function of the ER lead to accumulation of misfolded proteins and altered calcium homeostasis within the cell that ultimately activates the ER stress response pathway. In response to ER stress, the unfolded protein response is initiated by the activation of 3 independent pathways: PKR-like ER kinase (PERK), inositol-requiring enzyme 1 (IRE1), or activating transcription factor 6 (ATF6). PERK activation results in downstream phosphorylation of a subunit of eukaryotic initiation factor 2 (eIF2a) and upregulation of ATF4 (11–14). Under chronic ER stress, sustained ATF4 activation binds to the promoter of C/EBP-homologous protein (CHOP) and induces its transcription, which can lead to ER stress–induced apoptosis through regulation of BCL-2 family proteins and activation of caspase family proteins including cleaved caspase 3 and cleaved caspase 9 (15–17). Recently, the ER stress response has become an interventional target for a variety of cardiovascular diseases (18–21). Specifically, upregulation of PERK/ATF4 signaling and CHOP has been shown to contribute to cardiomyocyte dysfunction and progressive cardiac muscle loss in murine models of ischemic heart disease and heart failure (18, 22), while pharmacological inhibition or cardiomyocyte-specific deletion of CHOP was shown to prevent pathological cardiac remodeling (23). Regarding AAA disease, a recent study demonstrated increased expression of ER stress markers within human AAA tissue samples (24); however, the exact cellular mechanism by which aberrant ER stress activation contributes to progressive aortic dilation remains undefined.

In the present study, we provide experimental evidence that aberrant activation of the PERK/ATF4 stress response pathway directs VSMC-dysfunction and aortic aneurysm formation in 2 well-established murine AAA models (elastase-induced AAAs and angiotensin II–induced [AngII-induced] AAAs) and human aortic tissue samples. Using single-cell RNA-Seq (scRNA-Seq) transcriptome analysis of human AAA tissue, we identified upregulation of the PERK/ATF4 ER stress response in VSMCs resulting in alteration of apoptotic pathways within the aortic wall. Mechanistically, we demonstrate that TNF-α induces ATF4 activation within VSMCs through the PERK ER stress response, resulting in progressive apoptosis. Further targeted inhibition of the PERK/ATF4 pathway via VSMC-specific genetic depletion of PERK or pharmacological inhibition prevented AAA expansion and was associated with a preservation of VSMC function.

Overall, these findings identify the PERK/ATF4 ER stress response pathway as a critical regulator of VSMC loss during aneurysmal progression and demonstrate translational implications, as targeted manipulation of the PERK/ATF4 ER stress pathway is feasible to reduce AAA development.

## Results

### PERK/eIF2a/ATF4/CHOP signaling is increased in human and murine AAA tissues.

**Figure 1:**
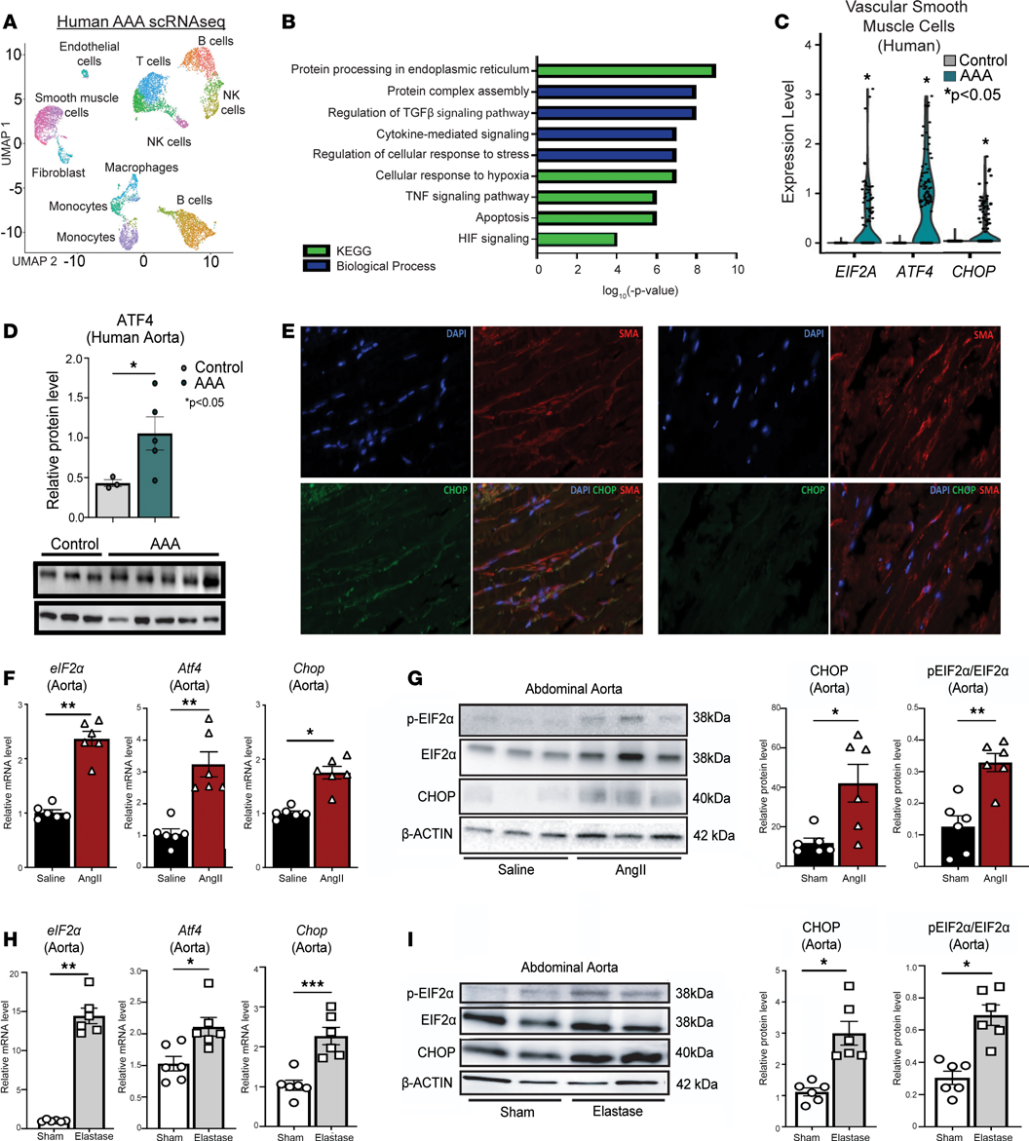
PERK/ATF/CHOP ER stress response is increased in VSMCs from human and murine AAAs. (**A**) Cluster analysis using the UMAP technique of single-cell RNA-Seq from human AAA and nonaneurysmal samples. (**B**) KEGG pathway and Gene Ontology biological process analysis of differentially expressed genes in VSMCs from AAA and nonaneurysmal aortas. (**C**) Violin plots of *EIF2A*, *ATF4*, and *CHOP* expression in VSMCs from AAA and nonaneurysmal aortas. (**D**) Protein abundance of ATF4 and β-actin determined by Western blot in human AAA and normal abdominal aortas (*n* = 3–5/group). (**E**) Representative immunofluorescence and quantification of CHOP (green) and smooth muscle α actin (SMA) (red) in human AAA and normal abdominal aortas (*n* = 3–5/group). Nuclei stained with DAPI are blue. Scale bars: 20 μm. (**F**) *Eif2a*, *Atf4*, *Chop* mRNA levels, relative to 18S, were determined by qPCR in murine abdominal aortas of mice infused with saline or AngII for 28 days (*n* = 6/group conducted in triplicate). (**G**) p-EIF2α, EIF2α, CHOP protein abundance in abdominal aortas of mice infused with saline or AngII (1000 ng/kg/min) determined by Western blot (*n* = 6 mice/group conducted in triplicate). (**H**) *Eif2a*, *Atf4*, and *Chop* mRNA levels, relative to 18s, were determined by qPCR in abdominal aortas of mice at day 14 after elastase or heat-inactivated elastase exposure for 30 minutes (*n* = 6 mice/group conducted in triplicate). (**I**) p-EIF2α, EIF2α, and CHOP protein abundance in the abdominal aortas of mice was determined by representative Western blot 14 days after elastase or heat-inactivated elastase exposure for 30 minutes (*n* = 6 mice/group). Bar graphs represent mean values. qPCR data represent experiments performed in triplicate. Data are shown as mean ± SE. Statistical analysis of data sets was performed by either Mann-Whitney *U* test or Kruskal-Wallis test (**C** and **F**–**I**). *P < 0.05; **P < 0.001.

To explore the role of VSMC dysfunction during AAA development, we first analyzed differential gene expression in human AAA and nonaneurysmal control VSMCs in our previously published human single-cell RNA-Seq dataset (25, 26) (Supplemental Table 1; supplemental material available online with this article; https://doi.org/10.1172/jci.insight.183959DS1). Within our human single-cell RNA-Seq analysis, the VSMC-related cluster contained high expression of contractile proteins *TAGLN*, *ACTA2*, and *MYL9*. Given that phenotypic and cellular alterations in VSMC biology have been suggested to play an important role in AAAs (27), we conducted pathway analysis on differentially expressed genes in the VSMC populations. Gene ontology demonstrated that VSMCs from AAA samples had upregulation of pathways involved in protein processing in the ER, protein complex assembly, cellular response to stress, and apoptosis in comparison with the same cellular subclusters in nonaneurysmal control samples (Figure 1, A and B). We then screened for alterations in known ER stress response genes in AAA VSMCs and found that *EIF2A*, *ATF4*, and *CHOP* expression were markedly elevated in the VSMC clusters in human AAA tissue in comparison with control samples (Figure 1C). This was further confirmed on Western blotting and immunofluorescence of human AAA tissues, which demonstrated elevated ATF4 in human AAA tissue and CHOP in human AAA VSMCs compared with control (Figure 1, D and E, and Supplemental Figure 1, A and B). Gene ontology demonstrated that VSMCs with upregulation of *EIF2A*, *ATF4*, and *CHOP* expression had corresponding upregulation of pathways involved in ER stress, smooth muscle cell migration, cellular death markers, and inflammation (Supplemental Figure 1C).

Given the elevated expression of *PERK/ATF4/CHOP* in VSMCs from human AAA tissue, we examined ER stress activation in murine VSMCs using an established AngII-induced murine model (28). Specifically, male C57BL/6J mice were injected i.p. with a single dose of an adeno-associated virus (AAV) vector expressing the mouse D377Y gain-of-function proprotein convertase subtilisin/kexin type 9 (PCSK9), which resulted in sustained hypercholesterolemia as described previously (28). Following this, mice were fed a saturated fat enriched diet for 6 weeks and received either saline or AngII infusion (1000 ng/min/kg) for the last 4 weeks. The use of a saturated fat diet and AngII infusion to induce AAAs recapitulates important clinical risk factors including dyslipidemia and hypertension. Consistent with data from human aortic tissue, murine AAA VSMCs displayed significantly elevated *eIF2a*, *Atf4*, and *Chop* gene and protein expression (Figure 1, F and G). To further confirm the importance of ER stress activation in VSMC pathophysiology during AAA development, we employed a second murine model of AAAs utilizing topical elastase treatment, which results in nondissecting AAA formation (29, 30). At day 14, following elastase application, there was marked upregulation of gene and protein expression of *eIF2a*, *Atf4*, and *Chop* in VSMCs from elastase-induced AAA mice compared with sham operated control animals (Figure 1, H and I). Collectively, these results suggest that upregulation of PERK/ATF4/CHOP ER stress response pathway may play a causative role in VSMC dysfunction in both human and murine AAAs.

### TNF-α induces ATF4/CHOP activation in VSMCs, resulting in increased VSMC apoptosis.

**Figure 2:**
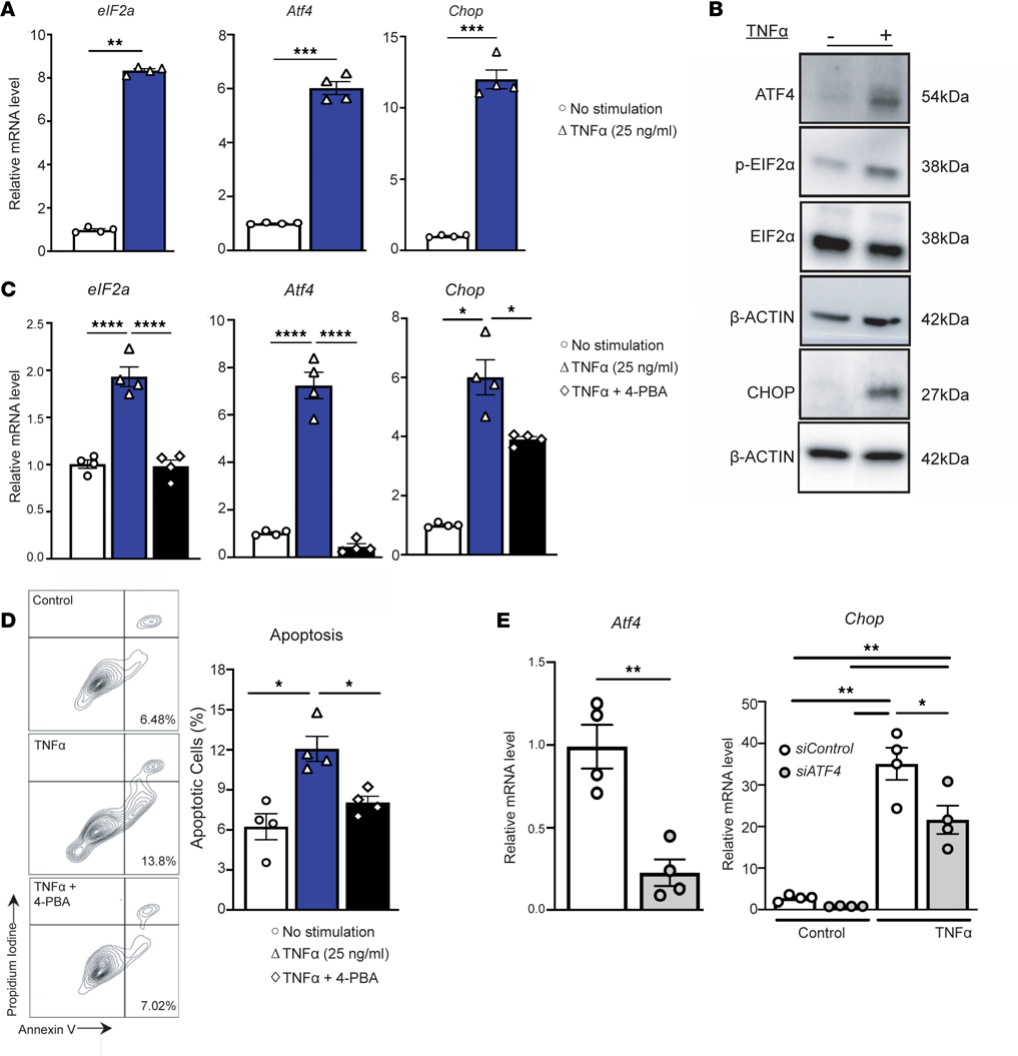
TNF-α induces ATF4/CHOP ER stress signaling and apoptosis in vascular smooth muscle cells. (**A**) MOVAS cells were treated with TNF-α (25 ng/mL) for 12 hours, and *eIF2a*, *Atf4*, and *Chop* mRNA levels, relative to 18S, were determined by qPCR. (**B**) MOVAS cells were treated with TNF-α (25 ng/mL) for 12 hours and p-EIF2α, EIF2α, ATF4, and CHOP protein abundance were determined by Western blot. Representative Western blotting is shown for treatment conditions and proteins of interest. (**C**) MOVAS cells were treated with TNF-α (25 ng/mL) and 4-PBA (3mM) for 12 hours, and *eIF2a*, *Atf4*, and *Chop* mRNA levels, relative to 18S, were determined by qPCR. (**D**) MOVAS cells were treated with TNF-α (25 ng/mL) and 4-PBA (3 mM) for 12 hours, and apoptosis was quantified by flow cytometry with annexin V and Propidium Iodine staining. (**E**) MOVAS cells were transfected with siControl or siATF4 (30 nM). After 48 hours, cells were serum starved in Opti-MEM for 24 hours. Depletion of *Atf4* was confirmed by qPCR analysis. Cells were then stimulated with TNF-α (25 ng/mL) for 12 hours, and *Chop* mRNA levels, relative to 18S, were determined by qPCR. Bar graphs represent mean values from at least *n* = 4 independent experiments, with individual data points representing independent experiments. Data are represented as mean ± SEM. 2-way ANOVA with Newman-Keuls multiple-comparison test (**C**–**E**). Mann-Whitney *U* test (**A**). *P < 0.05; **P < 0.01; ***P < 0.001; ****P < 0.0001.

Our scRNA-Seq data in human AAA tissues showed that in addition to activation of ER stress response pathways during AAA development, VSMCs also display elevations in TNF-α intracellular signaling (Figure 1B). Prior investigations have demonstrated that in vivo blocking of TNF-α using either a pharmacological agent (infliximab) or genetic depletion limits AAA development (infliximab) or genetic depletion (31, 32). However, the mechanism regarding why inhibition of TNF-α prevents AAA development and specifically its impact of VSMCs has remained undefined. Separately, additional studies have demonstrated that aberrant TNF-α signaling can mediate activation of VSMC dysfunction via ER stress pathways and VSMC calcification resulting from induction of mineralization and osteoblast differentiation of VSMCs (21). To more fully examine the impact of TNF-α signaling, ER stress activation, and VSMC dysfunction during AAA development, murine aortic VSMCs were isolated and stimulated with TNF-α which resulted in significant upregulation and activation of phosphorylated *eIF2a*, *Atf4*, and *Chop* at both the RNA and protein level (Figure 2, A–C). Further TNF-α stimulation, promoted VSMC apoptosis with significant elevation of annexin V staining in vitro. However, TNF-α–induced VSMC apoptosis was prevented with coadministration of an ER stress inhibitor 4-phenyl butyric acid (4-PBA) (Figure 2D). 4-PBA is widely recognized as a ER stress inhibitor because of its ability to act as a chemical chaperone in which the hydrophobic regions of 4-PBA interact with unfolded proteins in order to promote proper protein folding (33). As such 4-PBA prevents the accumulation of unfolded protein aggregates which are key stimuli/ligands that activate the 3 main ER stress pathways (PERK, IRE1, or ATF6) and subsequently reduces ER stress (34). To determine the specific ER stress pathway primarily responsible for TNF-α–induced VSMC apoptosis, we performed siRNA knockdown experiments of known effectors involved in the ER stress response. We utilized our scRNA-Seq findings to prioritize our analysis of candidate proteins, which included *EIF2a* and *ATF4*. We began our siRNA screen with ATF4, which demonstrated one of the most significant elevations of downstream ER stress effectors in VSMC in human AAA tissues (Figure 1C). Knockdown of ATF4 using siRNA, with 78% reduction in ATF4 expression, significantly prevented TNF-α–induced *Chop* expression (Figure 2E). Taken together, these results suggest that TNF-α–induced VSMC apoptosis is driven by activation of the ATF4/Chop ER stress response within in VSMCs.

### Pharmacological inhibition of ER stress activation via 4-PBA prevents AAA formation and decreases VSMC apoptosis.

**Figure 3:**
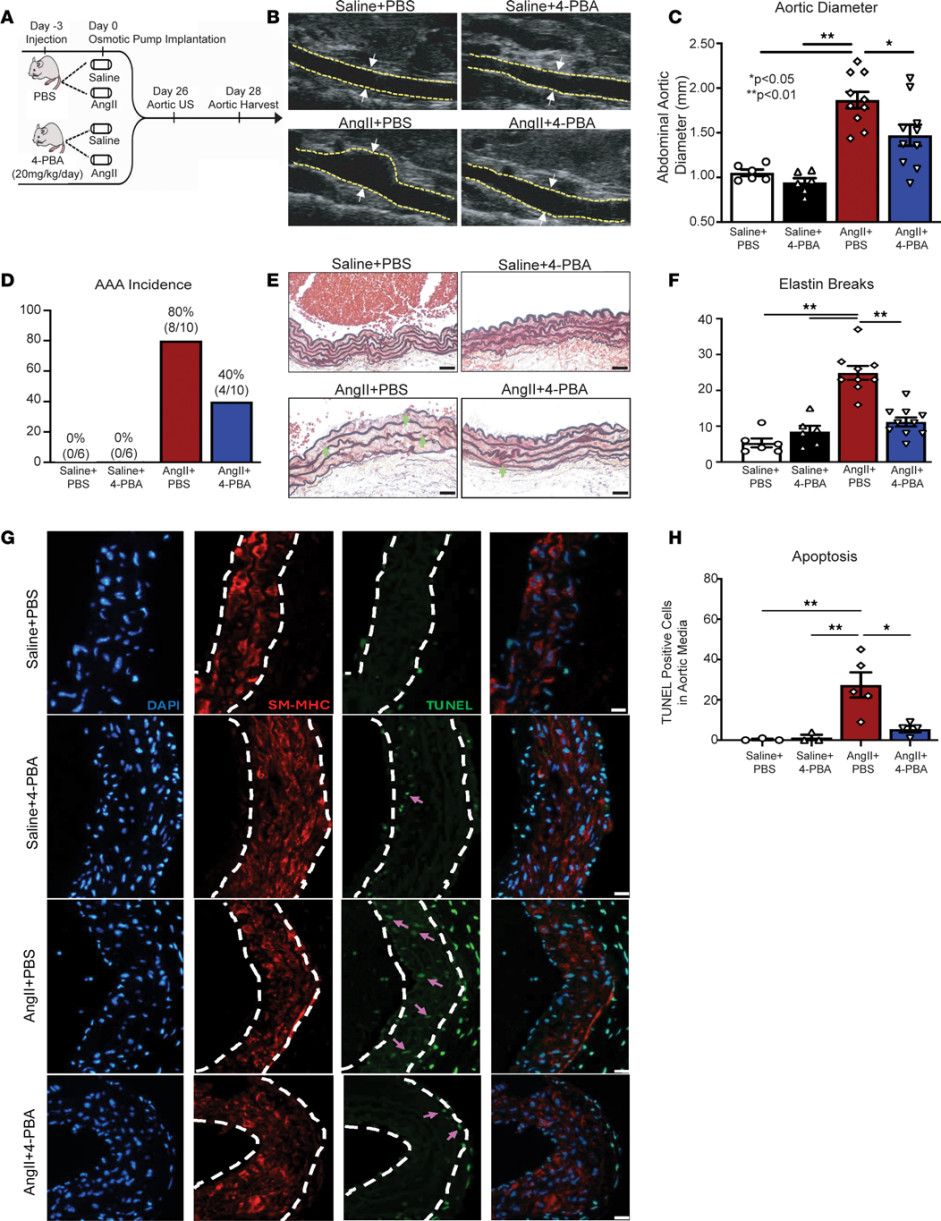
Pharmacological inhibition of ER stress response via 4-PBA prevents AAA formation. (**A**) Experimental design of ER stress response inhibition (4-PBA) in murine AAA model. WT mice were fed high-fat diet for 6 weeks and infused with saline or AngII infusion (1,000 ng/min/kg) for 4 weeks. During this period, mice were randomized to receive either PBS or 4-PBA (20 mg/kg/day) injection. (**B**) Representative ultrasound images of the abdominal aorta at day 28 in WT mice that received either saline or AngII infusion with or without 4-PBA treatment. Yellow dotted lines represent aortic border and arrows represent aortic diameter. (**C** and **D**) Maximal abdominal aortic diameter and aneurysm incidence as determined by ultrasound measured by 2 observers in WT mice infused with either saline or AngII with or without 4-PBA administration (*n* = 6–10 per group). (**E**) Representative Movat’s staining of abdominal aortic sections and associated analysis showing elastin lamina structure in AngII + 4-PBA mice compared with AngII + PBS mice. Scale bar: 50 μm; arrows represent elastin fragmentation (*n* = 6–10 per group). (**F** and **G**) Representative aortic sections for DAPI^+^ cells (blue), smooth muscle myosin heavy chain^+^ (SM-MHC^+^) cells (red), and TUNEL^+^ cells (green) staining in the media of suprarenal abdominal aortas (*n* = 5/group). Pink arrows correspond to TUNEL^+^ cells, and white dotted lines correspond to region of aortic media. Scale bars: 20 μm. Bar graph is the number of TUNEL^+^ cells per section averaged over 3–5 sections per animal. Data are represented as mean ± SEM. ANOVA with Newman-Keuls multiple-comparison test (**C**, **E**, and **G**). *P < 0.05; **P < 0.001.

Given that activation of the PERK/ATF4/Chop pathway was increased in human and murine AAAs and promoted VSMC apoptosis in vitro, we investigated the translational potential of ER stress inhibition in regulating AAA formation. To pharmacologically inhibit the ER stress response pathway, we chose to administer 4-PBA because of its ability to inhibit the ER stress response pathway, and it has been previously approved by the Food and Drug Administration for use in humans for the treatment of a variety of diseases (35). As shown in the schematic in Figure 3A, C57BL/6J mice were randomized to receive daily injection of 4-PBA (20 mg/kg) or PBS control starting 3 days prior to AngII or saline pump implantation with injections continuing throughout the 28-day infusion period. ER stress inhibition resulted in a marked reduction in AAA incidence and diameter (Figure 3, B–D). Histologic analyses showed that aortas from AngII-infused + 4-PBA–treated mice maintained a normal aortic architecture with a preserved smooth muscle cell layer and reduced elastic fiber fragmentation (Figure 3E and Supplemental Figure 1D). Our in vivo data further show that 4-PBA ER stress inhibition resulted in decreased VSMC apoptosis as measured by TUNEL staining (Figure 3, F–H).

**Figure 4:**
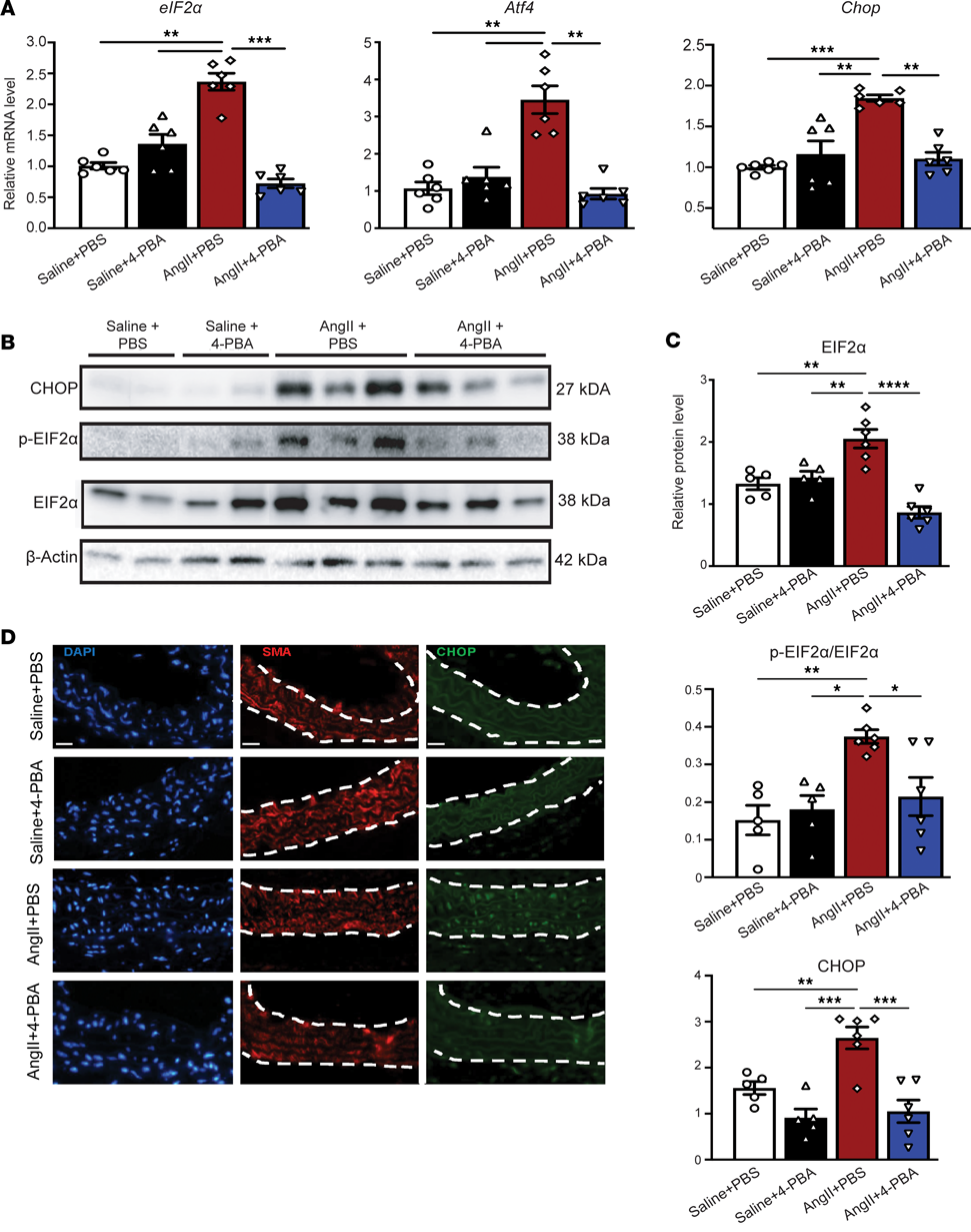
In vivo 4-PBA administration prevents ER stress response activation within aortic wall. (**A**) *eIF2a*, *Atf4*, and *Chop* mRNA levels, relative to 18S, were determined by qPCR in murine suprarenal abdominal aortas of C57BL/6J mice injected i.p. with AAV-Pcsk9.D377Y and infused with saline or AngII (1000 ng/kg/min) with or without 4-PBA (20 mg/kg/day) injection (*n* = 6/group). qPCR data represent experiments performed in triplicate. (**B** and **C**) p-EIF2α, EIF2α, and CHOP protein abundance in the suprarenal abdominal aortas of C57BL/6J mice injected i.p. with AAV-Pcsk9.D377Y and infused with saline or AngII (1000 ng/kg/min) with or without 4-PBA (20 mg/kg/day) injection by Western blot (*n* = 6/group). (**D**) Representative immunofluorescence staining of smooth muscle α actin (SMA) (red), CHOP (green), and DAPI (blue) in the aortic wall of suprarenal abdominal aortas. Dashed line represents border of aortic media. Scale bars: 20 μm. Data are presented as the mean ± SEM. 2-way ANOVA with Newman-Keuls multiple-comparison test (**A** and **B**). *P < 0.05; **P < 0.01; ***P < 0.001; ****P < 0.0001.

To further determine the effect of 4-PBA administration on AAA development, VSMCs were isolated on day 28 to analyze for ER stress activation. Mice that received AngII + 4-PBA treatment displayed a significant reduction in *Eif2a*, *Atf4*, and *Chop* gene and protein expression (Figure 4, A–D). The protective role of 4-PBA ER stress inhibition in AAA was further confirmed in the elastase-induced AAA model. Consistently, mice who received elastase + 4-PBA had a significant reduction in AAA diameter and ER stress pathway activation in comparison with elastase control mice (Supplemental Figure 1, E and F). Fourteen days after elastase exposure, there was no noticeable difference in body weight or blood pressure. Taken together, these findings suggest that activation of the PERK/EIF2A/ATF/CHOP pathway contributes to AAA development, and inhibition abrogates aneurysm formation by decreasing VSMC apoptosis.

### PERK depletion in VSMCs prevents AAA development in mice.

**Figure 5:**
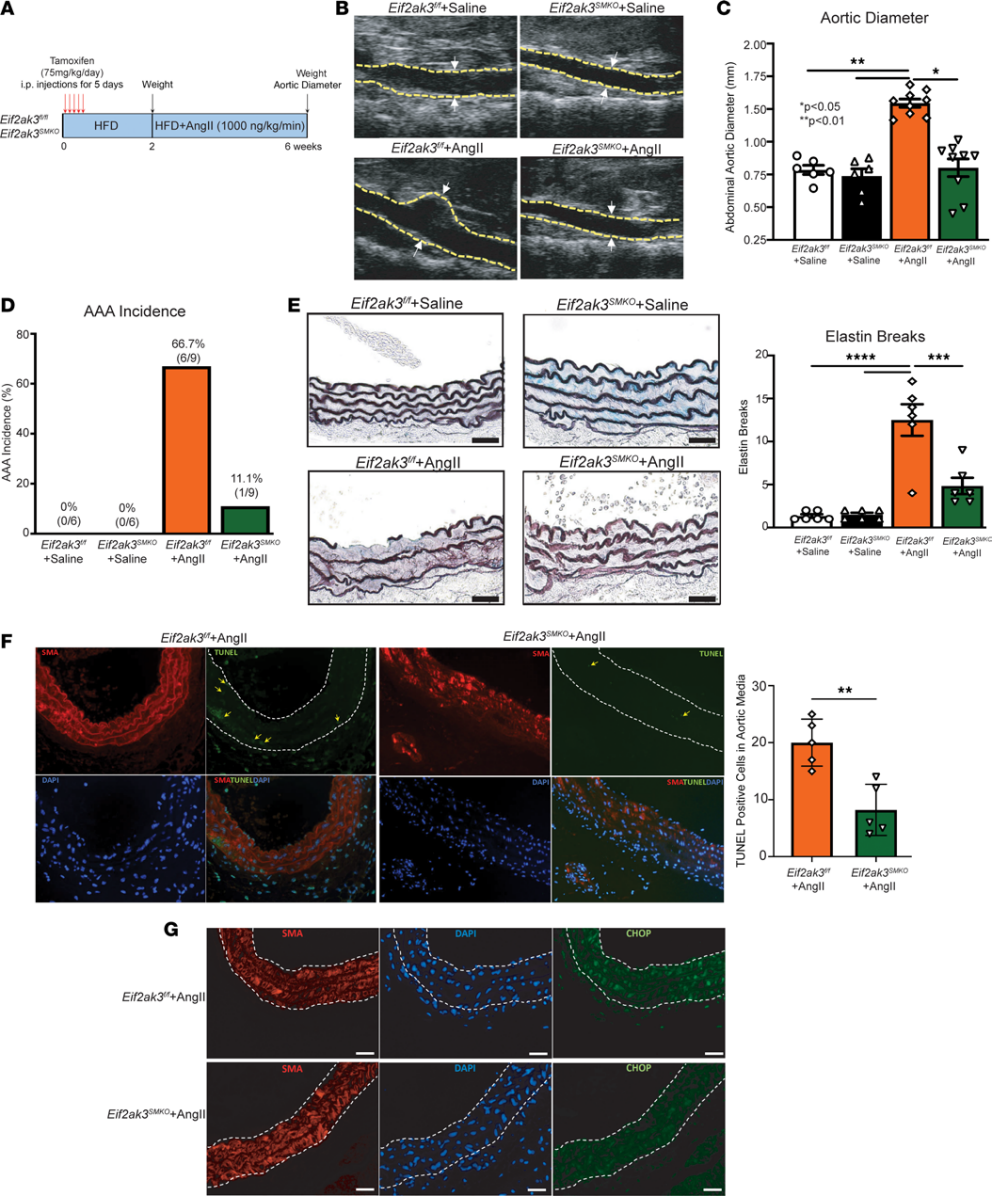
VSMC-specific PERK depletion prevents AngII-induced AAA development. (**A**) Schematic model. Ten-week-old male *Eif2ak3^fl/fl^* and *Eif2ak3^SMKO^* mice were injected with an AAV PCSK9 viral vector, and high-fat diet was initiated for 6 weeks. At week 2, mice had implantation of an osmotic pump for infusion of AngII (1,000 ng/kg/min) for a total of 4 weeks. (**B**) Representative ultrasound images of the abdominal aorta at day 28 in *Eif2ak3^fl/fl^* and *Eif2ak3^SMKO^* mice that received either saline or AngII infusion. Yellow dotted lines represent aortic border and arrows represent aortic diameter. (**C** and **D**) Maximal abdominal aortic diameter and aneurysm incidence as determined by ultrasound measured by 2 observers in *Eif2ak3^fl/fl^* and *Eif2ak3^SMKO^* infused with either saline or AngII (*n* = 6–9/group). (**E**) Representative Movat’s staining of abdominal aortic sections and associated analysis showing elastin lamina structure in *Eif2ak3^fl/fl^* and *Eif2ak3^SMKO^* infused with either saline or AngII. Scale bar: 50 μm. (**F**) Representative staining for smooth muscle α-actin (SMA) (red), TUNEL (green), and DAPI (blue), and quantification of the TUNEL^+^ cells in the media of suprarenal abdominal aortas (*n* = 6/ group). Yellow arrows correspond to TUNEL^+^ cells. Dotted line traces aortic media. Bar graph shows the number of TUNEL^+^ cells per section averaged over 3–5 sections per animal. Magnification, 50×. (**G**) Representative immunofluorescence staining of SMA (red), CHOP (green), and DAPI (blue) in the aortic wall of suprarenal abdominal aortas. Dashed line traces border of aortic media. Scale bars: 20 μm. Data are represented as mean ± SEM. *P < 0.05; **P < 0.01, ***P < 0.001; ****P < 0.0001, ANOVA with Newman-Keuls multiple-comparison test (**C** and **E**). Mann-Whitney *U* test (**F**).

Since global pharmacological inhibition of ER stress prevented AAA formation, we examined the effects of VSMC-specific genetic deletion of the PERK/ATF4/CHOP pathway on AAA development in a murine model. To evaluate the role of PERK in VSMCs in AAA development, we generated mice with a VSMC-specific deletion of PERK (gene symbol *Eif2ak3*) by crossbreeding Perk-floxed mice (*Eif2ak3^fl/fl^*) with *Myh11-CreER^T2^* mice; this was followed by tamoxifen induction to create a Eif2ak3 KO (*Eif2ak3^SMKO^*). The deletion of PERK (*Eif2ak3*) in *Eif2ak3^SMKO^* VSMCs was confirmed by PCR and Western blotting (Supplemental Figure 2, A and B). After crossbreeding and tamoxifen induction, *Eif2ak3^SMKO^* and *Eif2ak3^fl/fl^* mice were used for the AngII-induced AAA model (Figure 5A). Following 4 weeks of AngII (1,000 ng/kg/min) infusion, body weight and plasma lipid profiles were comparable between the 2 groups. Nevertheless, VSMC-specific *Eif2ak3* deficiency significantly decreased AAA diameter and incidence (Figure 5, B–D). Histologic analyses showed that aortas from *Eif2ak3^SMKO^* +AngII mice maintained a normal aortic architecture with preserved elastin lamin (Figure 5E). Furthermore, apoptotic cells within the aortic media were markedly reduced in *Eif2ak3^SMKO^* mice following AngII infusion compared with control tissues (Figure 5F). CHOP expression was analyzed to determine levels of ER stress activation within the aortic wall, demonstrating markedly reduced CHOP expression within *Eif2ak3^MKO^* mice following AngII therapy (Figure 5G). Overall, these data confirm the importance of the PERK/EIF2A/ATF/CHOP pathway within smooth muscle cells, which drives VSMC apoptosis during AAA development, and that targeted inhibition of the PERK pathway can prevent AAA development.

## Discussion

**Figure 6:**
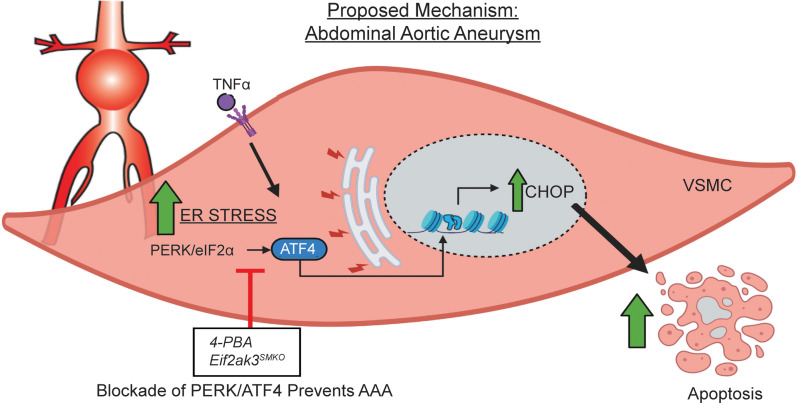
Schematic model of the pathological role of ER stress activation within VSMC during AAA development.

VSMC dysfunction is known to be an important factor contributing to AAA pathogenesis, as prior work has demonstrated that loss of VSMC within the aortic wall leads to aneurysm initiation, progression, and eventual rupture. However, the etiology behind this progressive VSMC apoptosis during AAA development remains unclear. Herein, using 2 murine models (AngII-induced AAA model and elastase AAA model) and human AAA tissue samples, we identified that pathological activation of the PERK/eIF2α/ATF4 ER stress response promotes VSMC apoptosis during AAA development. Mechanistically, elevated levels of TNF-α signaling within the aortic wall trigger upregulation of the PERK/eIF2α/ATF4 arm of the ER stress response, which results in Chop expression and VSMC apoptosis. Ultimately, manipulation of this pathway, by use of either a VSMC-specific genetic model (*Perk^fl/fl^ Myh11-CreERT2*) or with pharmacological inhibition, inhibited apoptosis and decreased aortic dilation, thereby offering promise as an AAA translational therapy (Figure 6).

Recently, there have been growing investigations implicating a pathological role of ER stress in a variety of cardiovascular disease states, including ischemic heart disease, hypertension, stroke, atherosclerosis, and heart failure (36–38). Regarding aortic aneurysmal disease, broad pharmacological agents that behave as promiscuous paninhibitors of the ER stress pathway have been shown to decrease aortic inflammation (39, 40). Additionally, global deletion of CHOP led to reduced aortic dilation (41–43). Separately, investigations by Zhao et al. demonstrated that genetic depletion of XBP-1 exacerbated AAA development via alteration of cell phenotype and inflammation within the aortic wall (44). However, these preceding studies failed to investigate the specific role of each unique branch within the ER stress response as well as the pathological activators of the ER stress pathway. Furthermore, these studies failed to detect cell-specific involvement of the pathological ER stress pathway.

Herein, we demonstrate that human AAA VSMCs display upregulation of the PERK/ATF4 ER stress response pathway and that this is likely mediated through increased TNF-α signaling within the aortic wall. Inflammatory cytokine signaling, particularly expression of IL-Iβ, TNF-α, IL-12, and IL-23, have been shown to be important drivers of AAA progression, and inhibition of many of these cytokines can prevent aneurysm formation (31, 45–47). Samples from patients with AAA have consistently shown elevation of TNF levels in both plasma and aneurysm wall tissue samples, thereby suggesting that TNF plays a significant role in the pathogenesis of the disease (47, 48). The main source of TNF-α within aortic aneurysmal disease has been shown to be infiltrating macrophages within the arterial wall (49). Studies have confirmed that inhibiting TNF-α can limit AAA progression by multiple mechanisms, including reducing proinflammatory cytokines, decreasing adhesion molecule expression, and limiting macrophage infiltration. Further support demonstrates that a lack of functional TNF-α in mice attenuates CaCl_2_-induced aneurysm development (31, 50). Separately, elevated levels of TNF-α have been linked to aberrant activation of the ER stress pathway in fibroblasts, endothelial cells, and macrophages in a variety of disease states (51–54). Interestingly, TNF-α signaling does not produce uniform activation of all 3 ER stress pathways across different cell populations. Instead, it appears that TNF-α signaling selectively activates individual ER stress pathways in a disease- and cell-specific manner. Masuda et al. demonstrate that vascular smooth muscle calcification arising during chronic kidney disease is mediated by increased reactive oxygen species and TNF-α–inducing PERK signaling but no difference in ATF6 or IRE1 activation (21). Our findings support this selective ER stress pathway activation during AAA development, since VSMCs from human and murine aneurysmal disease display elevated levels of TNF-α signaling as well as PERK/eIF2α/ATF4 signaling, without corresponding changes in other ER stress pathways. The increase in TNF-α signaling altered levels of VSMC apoptosis in vitro that were dependent on the ATF4 pathway, as siRNA depletion of ATF4 prevented TNF-α–mediated VSMC dysfunction.

Given the importance of the PERK/eIF2α/ATF4 ER stress pathway, we investigated if inhibition of the ER stress response pathway altered VSMC function and AAA development. Administration of a pharmacological inhibitor (4-PBA) led to reduced abdominal aortic dilation, improved vascular wall remodeling, and decreased loss of VSMC inflammation. Although pharmacological inhibition with 4-PBA provides important mechanistic insight, 4-PBA can inhibitor other ER stress pathways (IRE-1/XBP and ATF6) as well as have off-target effects on histone deacetylases (55). To provide increasing mechanistic detail, VSMC-specific PERK-KO animals were created. These VSMC-specific PERK-KO mice demonstrated a reduction in AAA formation and decreased VSMC apoptosis. Prior work by ourselves and others has demonstrated that, within atherosclerosis, activation of the ER stress pathway can contribute to VSMC apoptosis and plaque progression (56), while treatment with an ER stress inhibitor, tauroursodeoxycholic acid, decreased medial VSMC loss and atherosclerosis (57, 58). Of note within the current study, the VSMC-specific PERK KO had a more profound inhibition on AAA development than 4-PBA pharmacological inhibition. This result is likely multifactorial, as 4-PBA is accepted as an ER stress inhibitor; it inhibits all pathways within the ER stress response by functioning as a chemical chaperone and preventing the buildup of misfolding proteins aggregates. Therefore, the 4-PBA chemical is not uniquely specific to PERK/ATF4 pathway and therefore not completely potent. In contrast, our VSMC-specific PERK–genetic KO mouse *Eif2ak3^SMKO^* displayed a profound reduction in the PERK receptor, thereby indicating a more specific effect on the PERK/ATF4 pathway and VSMC apoptosis.

Alternative to VSMC apoptosis, activation of the ER stress pathway can affect smooth muscle cell phenotype (56, 59). Indeed, we have previously shown that, during atherosclerotic plaque formation, exposure of smooth muscle cells to free cholesterol or oxidized LDL can result in abnormal activation of all 3 pathways within the ER stress response and, in turn, leads to alteration of smooth muscle cell phenotype in cholesterol rich VSMCs (56, 59). Inhibiting cholesterol transport from the plasma membrane to the ER prevents cholesterol-induced activation of the ER stress response. In vivo, cholesterol-induced phenotypic switching was prevented by global pharmacological or genetic inhibition of the ER stress response in a Perk-dependent and Perk-independent manner (59, 60). Given that AAA development is not dependent on cholesterol accumulation, the role of ER stress–driven VSMC phenotypic conversion in aneurysmal disease progression remains to be defined. Ultimately, the ability to modulate VSMC apoptosis and cellular function after vascular injury is an attractive therapeutic strategy for AAA management. Manipulation of the ER stress response has been shown to be effective in the treatment of multiple cancers. Indeed pharmacological inhibition of the ER stress response can result in dose-dependent inhibition of cell proliferation, invasion, and cell migration (61–63). More recently, Wang et al. have designed a nanocarrier linked to a PERK inhibitor that was able to prevent stenosis, thrombosis, and VSMC phenotypic switch in a murine model (64, 65). Lastly, pharmacological inhibition of ER stress activation is currently undergoing phase II clinical trials for the treatment of hypertension (NCT06025630). Despite these promising results of investigations for cardiovascular disease, the role of PERK/eIF2α/ATF4 inhibition in VSMC function during AAA development remains to be fully defined. Our results demonstrate the PERK/eIF2α/ATF4 activity within VSMCs may be an attractive therapeutic target for AAAs, as it can prevent smooth muscle dysfunction and decrease pathological vascular remodeling.

Although this study provides valuable insight into the mechanisms behind VSMC dysfunction and homeostasis in AAA development, some limitations must be addressed. First, the human AAA samples may not represent the entire etiology of AAA formation, as they are taken from end-stage disease. Therefore, the timing of ER stress elevation during human AAA dilation remains unknown. Secondly, within our investigation, we chose to use the Myh11-CreERT2 transgenic mouse generated for smooth muscle cell–specific deletion of PERK. This transgenic mouse line has been extensively used for SMC-specific lineage tracing (66–69). The major limitation of this mouse is that the Myh11-CreERT2 transgene is located on the Y chromosome, which only allows for the study of male mice. Therefore, we are currently working to utilize smooth muscle–specific Cre drivers (70, 71) to further investigate the effect of PERK KO in female mice and the effect on the gut microbiome. Lastly, within our investigations, 4-PBA administration or genetic depletion of the PERK pathway occurred prior to initiation of AAA dilation. Within human aortic disease, patients present with a preestablished aortic aneurysm, and as such, the ability of a pharmacological therapy to prevent aortic initiation and contribute to the regression of existing AAAs is important in consideration for translational therapy.

In conclusion, our study provides important mechanistic evidence that PERK/eIF2α/ATF4 signaling regulates VSMC dysfunction and apoptosis during human and murine AAA development. Targeting the PERK/eIF2α/ATF4 pathway affords the opportunity to prevent pathological loss of VSMCs within the aortic wall and potentially limit AAA progression and rupture.

## Methods

### Sex as a biological variable.

Mice were maintained in the University of Michigan pathogen-free animal facility. Male mice were used for AAA experiments as detailed in the American Heart Association Council statement (72). Only male mice were used for these studies, as female mice do not adequately develop AAAs (72). C57BL/6J mice maintained on a normal diet (ND) (13.5% kcal fat; LabDiet, 5001) were purchased at 8–10 weeks from The Jackson Laboratory. *Eif2ak3^SMKO^* (*Eif2ak3^fl/fl^ Myh11-CreERT*2) control mice were obtained from Dianna Milewicz (University of Texas Health Science Center at Houston, Houston, Texas, USA) and were maintained in breeding pairs at the Unit for Laboratory Animal Medicine facilities. Of note, Dianna Milewicz initially created the *Eif2ak3^SMKO^* mice by breeding the *Eif2ak3^tm1.2Drc/J^* mice obtained from The Jackson Laboratory (strain no. 023066) to the SMMHC-iCreER^T2^ mouse (strain no. 036935). The *Eif2ak3^tm1.2Drc/J^* mice contain loxP sites that flank exons 7–9 in the *Eif2ak3* gene and were originally published by Zhang et al. (73). Primer sequences to confirm appropriate loss of target sequence include: forward primer, 5′-CCTTGGTTTCATCTAGCCTCA-3′; reverse primer, 5′-ATCCAGGGAGGGGATGAT-3′. Because the Cre transgene was inserted into Y chromosome (66), only male *Eif2ak3^fl/fl^ Myh11-CreERT*2 mice could be generated, and accordingly, only male mice were used in this study. The male *Eif2ak3^fl/fl^ Myh11-CreERT*2 mice were i.p. injected with either tamoxifen (MilliporeSigma, T5648, 75 mg/kg/day) or corn oil control for 5 consecutive days to generate VSMC-specific PERK knockout (*Eif2ak3^SMKO^*, *Eif2ak3^fl/fl^ Myh11-CreERT*2) and floxed control (*Eif2ak3^fl/fl^*, *Eif2ak3^fl/fl^ Myh11-CreERT*2) mice for the current study. Mice were allowed 9 days to clear the excess tamoxifen and were subjected to murine AAA models. Animals were housed in a barrier facility on a light/dark cycle of 14:10 hours (ambient temperature of 22°C) with free access to water, food (LabDiet, 5001), and bedding (Andersons Lab Bedding Bed-o’Cobs Combination). Animals underwent all procedures at 8–10 weeks of age.

### Production and injection of AAV vectors.

AAV vectors (serotype 8) were produced by the Viral Vector Core at the University of Pennsylvania (https://gtp.med.upenn.edu/core-laboratories-public/vector-core). These AAV vectors contained inserts expressing mouse PCSK9D377Y mutation (equivalent to human PCSK9D374Y gain-of-function mutation). Empty AAV vector (null AAV) was used as control. AAV vectors were diluted in sterile PBS (200 μL per mouse) and injected i.p. as reported previously (28, 74). Mouse inclusion and randomization were conducted as previously specified. Furthermore, mice had a predefined exclusion from the data analysis if plasma total cholesterol concentrations were < 250 mg/dL 3 weeks and < 500 mg/dL 6 weeks after PCSK9D377Y.AAV injection (28). Briefly, mice received injections of AAVs containing either a null insert or a mouse PCKS9 insert expressing D377Y mutation i.p. with 2 × 10^11^ genomic copies of AAV carrying a gain-of-function mutation of mouse Pcsk9. Immediately after AAV injections, normolipidemic mice were fed a diet containing saturated fat (TD.88137, Envigo; 0.2% cholesterol and 21.2% percent fat by weight) for 2 weeks, at which time they underwent implantation of mini osmotic pumps as described below. Body weights were determined prior to experimentation.

### Osmotic mini pump implantation and AngII infusion.

To induce AAAs, 8- to 10-week-old male C57BL/6J mice were injected with AAV and were started on a saturated fat diet as detailed above. Following 2 weeks of saturated fat diet feeding, mice were randomized to receive mini osmotic pumps (model 2004; Alzet) containing AngII (1,000 ng/min per kilogram; H-1706, Bachem) or saline were implanted s.c. in the neck region of anesthetized mice following a protocol described previously (75). Briefly, mice were anesthetized in a closed chamber with isoflurane (3%) in oxygen for 2–5 minutes until immobile. Each mouse was then removed and taped on a heated (35°C–37°C) procedure board with isoflurane (1.0%–1.5%) administered via nosecone during minor surgery. Pumps were implanted s.c. on the right flank of each mouse that provided AngII or saline infusion for 28 days. Incisions were closed with surgical staples, and postoperative analgesia (buprenophine, Fidelis Pharmaceuticals, 0.05 mg/kg/12 hours, i.p.) was administered. For inhibitor studies, mice were randomized to receive every-other-day i.p. injection of GSK-J4 (10 mg/kg; Tocris, 4594) in 0.1% DMSO in PBS or of 0.1% DMSO in PBS alone. These injections began 3 days prior to mini osmotic pump implantation and continued for the 28-day duration.

### Elastase treatment model of aneurysm formation.

A murine elastase treatment model of AAA formation was used as described by Laser et al. (76). In brief, the infrarenal aorta was treated topically with 30 μL elastase reconstituted with normal saline (5 U/mg protein) or 30 μL heat-inactivated elastase (at 90°C for 30 minutes) as a control group. The topical application was accomplished by dropping the elastase on the anterior aorta from a 2 cm height for 5 minutes. Video micrometric measurements of aortic diameters were made in situ before perfusion, after perfusion, and before harvesting the aorta on day 14. Maximum infrarenal aortic diameter and ratio of treated versus untreated section of the aorta was calculated.

### ER stress inhibition with 4-PBA.

To evaluate the impact of ER stress inhibition on AAA development at the time of osmotic pump implantation mice were randomized to receive i.p. injection of either PBS control or 4-PBA 20 mg/kg/day for a total of 28 days. The chemical 4-PBA has gained widespread recognition as an “ER stress inhibitor” primarily because of its ability to act as a chemical chaperone (33). The main mode of action of 4-PBA involves interaction with hydrophobic regions of unfolded proteins in order to promote proper protein folding. Therefore, 4-PBA prevents the accumulation of unfolded protein aggregates, which are key stimuli/ligands that activate the 3 main ER stress pathways (PERK, IRE1, or ATF6), and subsequently reduces ER stress (34). Within the current investigation, we did not perform toxicology studies for 4-PBA, as these have already been conducted both for liver (77) and renal toxicology (78). Moreover, 4-PBA has already received approval from the Food and Drug Administration for use within humans. The prior research demonstrated that 4-PBA does not cause any hepatic or renal impairment even when administered at much higher doses than were administered by our protocol.

### Quantification of aortic pathologies.

For in vivo imaging of the abdominal aorta in mice, 2D (B-mode) ultrasound images were obtained 27 days after the implantation of osmotic pumps using a VisualSonics Vevo2100 imaging system with a mechanical transducer (MS400) from the University of Michigan Frankel Center for Physiology. Two independent investigators measured aortic diameters at systole with no significant interobserver or intraobserver variability.

At the completion of each murine aneurysm experiment (day 28), mice were deeply anesthetized with ketamine (100 mg/kg) and xylazine (20 mg/kg). At termination after blood collection, the right atrium was cut open, and saline was perfused through the left ventricle to remove blood in aortas. Subsequently, aortas were dissected and placed in either RNA later or 10% neutrally buffered formalin overnight at room temperature. After fixation, periaortic adventitia were removed thoroughly. Maximal outer diameters of suprarenal aortas were measured ex vivo as a parameter for AAA quantification using ImageJ software (NIH).

Necropsy was performed for mice that died during AngII infusion. Aortic rupture was defined as observation of blood clots in either the thoracic cavity (thoracic aortic rupture) or retroperitoneal cavity (abdominal aortic rupture). There was no difference in early rupture between treatment groups and genotypes.

### Histology/immunofluorescence.

For aortic histology, aortas were excised as above and fixed in formalin (10%) overnight before embedding in paraffin. Sections (5 μm) were stained with H&E, Verhoeff–van Gieson stain for elastin (Newcomer Supply, 9116B). Images were captured using Olympus BX43 microscope and Olympus cell Sens Dimension software.

For immunofluorescence, formalin-fixed, paraffin-embedded tissue slides obtained from patients with AAA, and aortic healthy control were heated for 30 minutes at 60°C, deparaffinized, and rehydrated. Slides were placed in 10 mM citric antigen retrieval buffer (pH.6.0) and heated at 95°C for 25 minutes in a steamer. After cooling, slides were blocked using 10% donkey serum for 30 minutes followed by 1:100 CHOP (Cell Signaling, L63F7), 1:250 anti–smooth muscle heavy chain I antibody (Abcam, ab133567), anti–murine antibodies SM22α (Abcam, ab103135), or anti–smooth muscle α actin (Invitrogen, 14-9760-82) for 1 hour at room temperature. Slides where then washed 3 times with PBS, incubated with 1:500 diluted Alexa Fluo 594–labeled donkey anti–mouse IgG secondary antibody (Invitrogen, A-21203) or 1:500 diluted Alexa Fluo 488–labeled donkey anti–rabbit IgG secondary antibody (Jackson ImmunoResearch, 711-545-152) for 45 minutes at room temperature. Slides were sealed with Fluoromount-G Mounting Medium containing DAPI (Sigma-Aldrich) after washes with PBS; this was done 3 times. Images were acquired using a Olympus Fluorescence Microscopy with cellSens Entry software. Images presented are representative of at least 3 biologic replicates. Control antibody staining is shown in Supplemental Figure 3.

### Cell culture.

Murine aortic smooth muscle cells (MOVAS, CRL-2797) were purchased from ATCC and cultured in SMC growth medium (ATCC, 30-2002) containing 10% FBS (ATCC) and 0.2 mg/mL G-418 at 37°C, 5% CO_2_ in a humidified cell culture incubator. Cells were stimulated with TNF-α (25 ng/mL) or 4-PBA (3mM) for 12 hours, and cells were collected for RNA analysis or annexin V staining as described below. VSMCs were used from passages 4–8 in all experiments.

### Flow cytometry analysis of apoptosis.

MOVAS cells were stimulated with TNF-α (25 ng/mL) or 4-PBA (3mM) for 12 hours. The cells were then trypsinized and stained with FITC Annexin V/PI (BioLegend, 640914) according to the manufacturer’s instructions, followed by flow cytometry analysis with Novocyte flow cytometer at the University of Michigan. The results were analyzed using FlowJo software (version 10.8.1, BD Biosciences).

### Apoptosis assay.

The DeadEnd Fluorometric TUNEL system (Promega, G3250) was used to detect cell apoptosis according to the manufacturer’s protocol. Briefly, to detect cell apoptosis in paraffin-embedded aortic tissue, the rehydrated tissue sections (5 μm thick) were incubated in 0.85% NaCl for 5 minutes at room temperature before fixation in 4% methanol-free formaldehyde solution for 15 minutes at room temperature. Next, the sections were permeabilized in Proteinase K solution (20 μg/mL) for 10 minutes at room temperature and fixed in 4% methanol-free formaldehyde solution for 5 minutes at room temperature, followed by incubation with equilibration buffer for 10 minutes at room temperature. The terminal deoxynucleotidyl transferase (TdT) reaction mixture was added to the aortic sections for 60 minutes with incubation at 37°C, followed by SSC buffer to stop the reaction. Slides were mounted with ProLong Gold Antifade Mountant with DAPI (Invitrogen, P36935), and the green fluorescence of apoptotic cells within the aortic wall was captured with an Olympus DP73 microscope. Quantification of the percentage of TUNEL^+^ cells in the media was performed with ImageJ software, and the average number of TUNEL^+^ cells per section determined across 3–5 sections for each mouse was calculated.

### siRNA transfection.

MOVAS were transfected with 30 nM siATF4 (Thermo Fisher Scientific, AM16708) or Silencer Select Negative Control siRNA (siControl, Thermo Fisher Scientific, 4390843) using Lipofectamine RNAiMAX Reagent (Invitrogen, 13778150) according to the manufacturer’s instructions.

### RNA analysis.

Total RNA extraction was performed using Trizol (Invitrogen) according to manufacturer’s instructions. RNA was then reversed transcribed to cDNA using iScript (Biorad). PCR was performed with 2× Taqman PCR mix using the 7500 Real-Time PCR System. Primers for *Eif2a* (Mm01289723_m1), *Atf4* (Mm00515324_m1), and *Chop* (Mm00492097_m1) were purchased from Applied Biosystems. 18S or GAPDH was used as the internal control. Data were then analyzed relative to 18s ribosomal RNA or GAPDH (2^ΔCt^). All samples were assayed in triplicate. The Ct values were used to plot a standard curve. Data are representative of 2–3 independent experiments were compiled in Microsoft Excel and presented using Prism software (GraphPad).

### Protein extraction and Western blot.

Cells were lysed in RIPA lysis buffer (Thermo Fisher Scientific, 89901) supplemented with the cOmplete EDTA-free protease inhibitor cocktail (Roche, 11873580001) and PhosSTOPTM phosphatase inhibitor (Roche, 4906845001). Human and mouse tissues were homogenized in T-PERTM tissue protein extraction reagent (Thermo Fisher Scientific, 78510). Cells or Tissues were lysed at 4°C for 30 minutes and centrifuged at 20,000 *g* for 15 minutes to remove insoluble debris. Protein extracts were resolved in 4%–20% SDS-PAGE gels and transferred to nitrocellulose membranes (BioRad, 1620115). Membranes were blocked in TBST containing 5% fat-free milk for 1 hour at room temperature and incubated with primary antibodies at 4°C overnight. After washing 3 times with 1×-TBST, membranes were incubated with secondary antibody (1:10,000 dilution, Li-Cor Bioscience) for 1 hour at room temperature. After 3 washes with 1×-TBST, bands were scanned using Odyssey Imaging System (Li-Cor Bioscience) and quantified with the LI-COR Image Studio Software. Primary antibodies against human ATF4 (catalog D4B8), murine ATF4 (catalog D4B8), murine p-EIF2α (catalog D9G8), murine EIF2α (catalog D7D3), murine CHOP (catalog L63F7), and β-actin (rabbit mAb 4970 and mouse mAb 3700) were purchased from Cell Signaling Technologies. Secondary antibodies included Goat Anti-Rabbit (catalog 32460) and Goat Anti-Mouse (catalog 32430) were purchased from Invitrogen.

### Human tissue.

Full-thickness aortic wall tissue specimens were collected from the infrarenal abdominal aorta from patients undergoing open aortic aneurysm repair or open aortobifemoral bypass. The aneurysmal samples were taken from the midportion of the aneurysmal sac. For control samples, aortic tissue was isolated from patients with atherosclerotic occlusive disease but with no history of aneurysmal disease at the time of open aortobifemoral bypass. Patient medical comorbidities are represented in Supplemental Table 1. All aortic samples were processed for both histology and protein/RNA analyses. For histology, human aortas were placed in formalin (10%) for 24 hours prior to paraffin embedding. Sections (5 μm) were stained with H&E or Masson’s trichrome stain for collagen deposition. Images were captured using an Olympus BX43 microscope and Olympus cell Sens Dimension software. For protein/RNA analysis, specimens were stored at –80°C for future protein and RNA analyses. For scRNA-Seq, a second cohort of samples was retrieved from the infrarenal abdominal aorta of patients undergoing open aortic aneurysm repair (*n* = 4) or open aortobifemoral bypass (*n* = 2). Patient medical comorbidities can be seen in Supplemental Table 2.

For scRNA-Seq analysis, we reanalyzed our previously published single cell dataset with Gene Expression Omnibus (GEO) accession no. GSE166676. For details on bioinformatic analysis and tissue processing please, see prior publications (25, 26).

### Statistics.

Data were analyzed using GraphPad Prism software version 6. Unless indicated otherwise, data are presented as mean ± SEM. All data were evaluated for normality and equal variance. For normally distributed data, 2-tailed Student’s *t* test was used to compare the difference between 2 groups, and 2-way ANOVA followed by Holm-Šidák post hoc analysis were used for comparison among 3 or more groups. For data that were not normally distributed, nonparametric tests, including Mann-Whitney *U* test, χ^2^ test, or Mantel-Cox test (survival percentage) were used to compare 2 groups. *P* < 0.05 was considered as statistically significant. All results are representative of at least 3 independent experiments. *P* < 0.05 were considered significant.

### Study approval.

Animal experiments were conducted following the *Guide for the Care and Use of Laboratory Animals* (National Academies Press, 2011) and were approved by the IACUC of the University of Michigan. The human aortic tissues used in this study were obtained from the he Frankel Cardiovascular Center (CVC) at the University of Michigan with the IRB HUM00098915 from the Human Research Protection.

### Data availability.

Values for all data points in graphs are reported in the Supporting Data Values file. Queries should be directed to and will be fulfilled by the corresponding author.

## Supplementary Material

Supplemental data

Unedited blot and gel images

Supporting data values

## Version 1 01/23/2025

Electronic publication
