# Supplementary material for: Inhibition of vascular smooth muscle cell PERK/ATF4 ER stress signaling protects against abdominal aortic aneurysms
Source: JCI Insight. 2025 Jan 23;10(2):e183959. doi: 10.1172/jci.insight.183959 (PMC11790032; doi:10.1172/jci.insight.183959)
Supplement: Supplemental data [file jciinsight-10-183959-s189.pdf]

Supplemental Figure 1

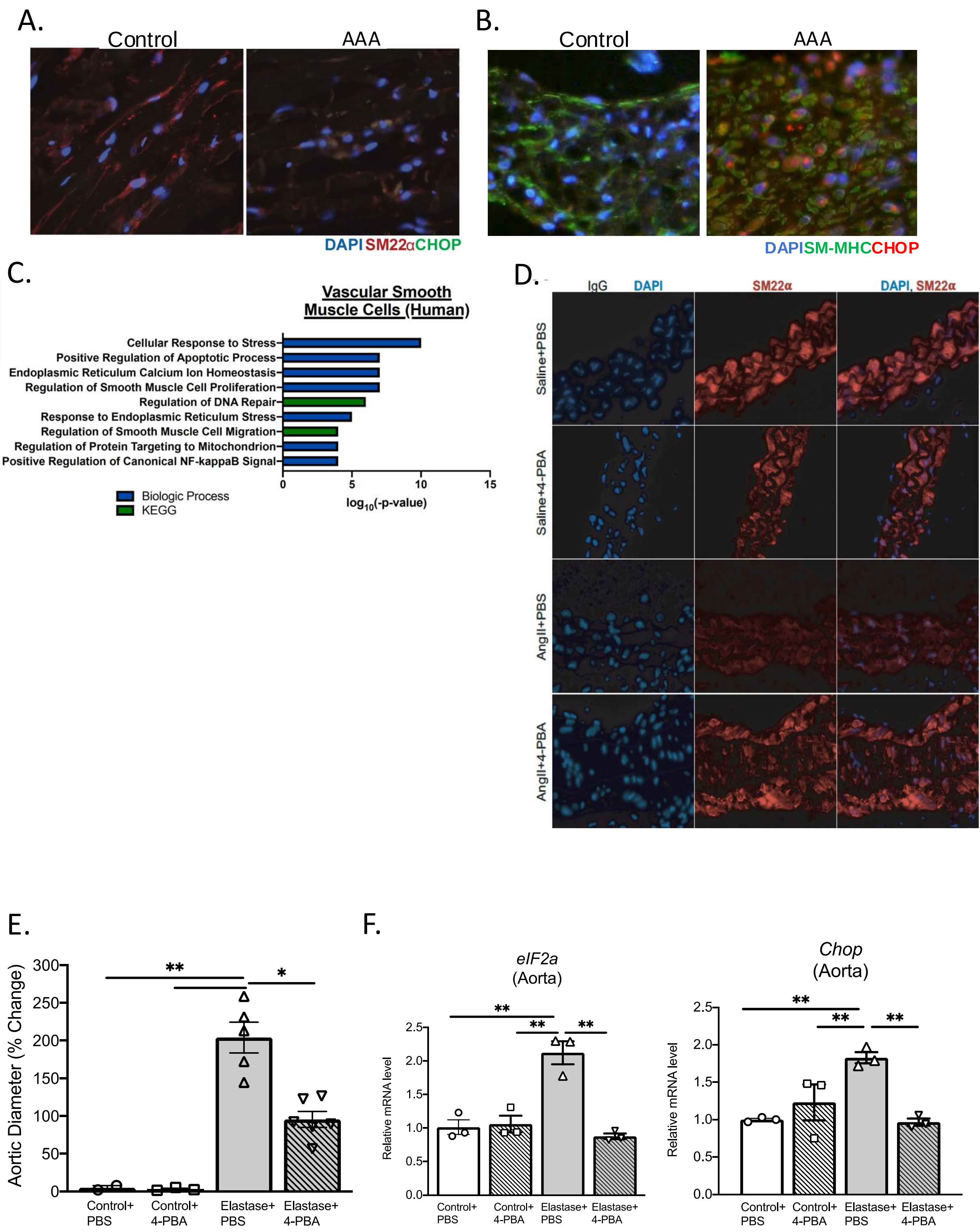

Supplemental Figure 2

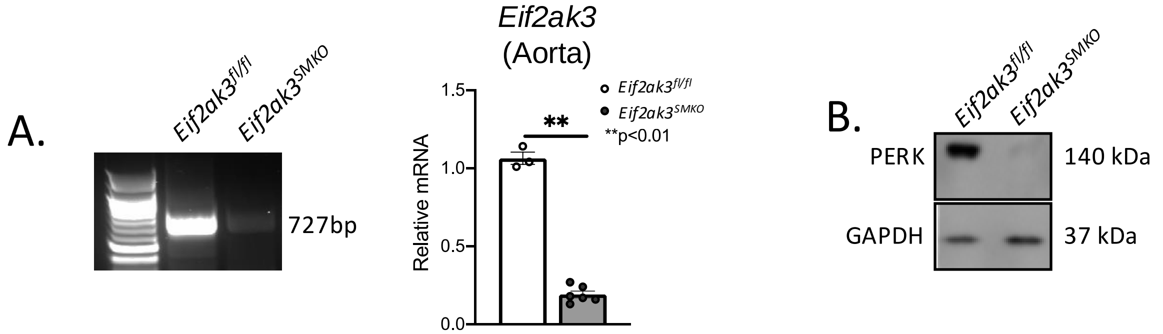

Supplemental Figure 3

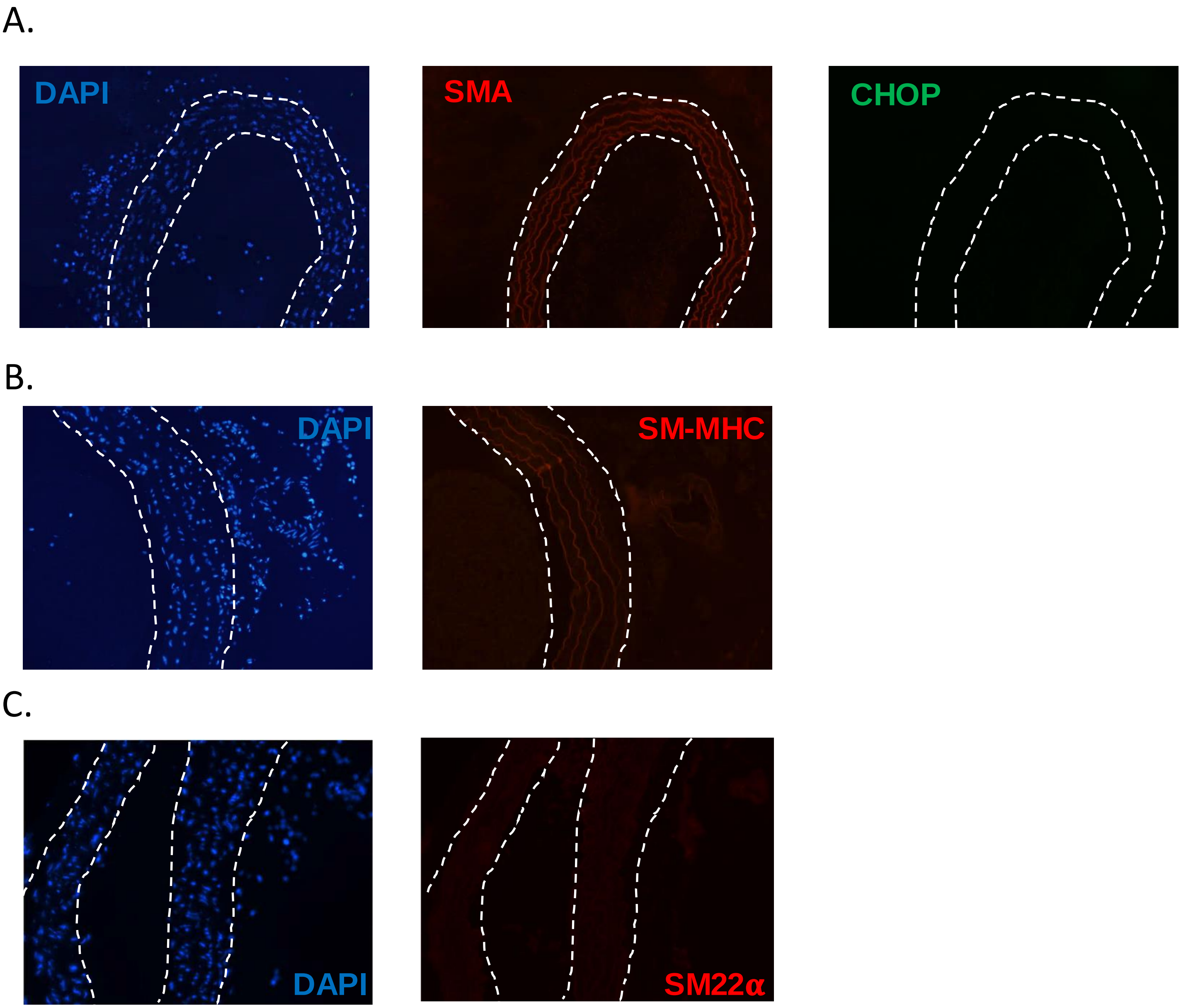

Supplemental Table I Human AAA and Atherosclerotic Tissue Cohort

Demographic	Control (n=3)	AAA (n=19)
Gender (% male)	2 (66.7)	15 (78.9)
Age (SD)	67.7 (7.5)	66.4 (7.3)
BMI (SD)	29.0 (6.3)	27.1 (4.9)
Aortic Diameter (SD, mm)	---	53.7 (12.8)
Tobacco abuse (prior/current)	2 (66.7)	17 (88.8)
Hypertension	2 (66.7)	17 (77.7)
Hyperlipidemia	2 (66.7)	12 (63.2)
Coronary artery disease	2 (66.7)	6 (31.5)
Diabetes	0 (0)	1 (5.2)
COPD	2 (66.7)	4 (21.0)
Renal Failure	0 (0)	1 (5.2)

BMI, body mass index; COPD, chronic obstructive pulmonary disease; SD, standard deviation

Continuous variable are summarized by mean (standard deviation) and categorical variables are summarized by N (%).

Supplemental Table II Human AAA and Atherosclerotic Single Cell RNA-Sequencing Cohort

Demographic	Control (n=2)	AAA (n=4)
Gender (% male)	2 (100)	2 (50)
Age (SD)	59.8 (2.1)	67.2 (15.5)
BMI (SD)	33.7 (4.0)	33.7 (6.0)
Aortic Diameter (SD, mm)	---	59.7 (3.3)
Tobacco abuse (prior/current)	2 (100)	2 (50.0)
Hypertension	2 (100)	2 (50.0)
Hyperlipidemia	2 (100)	2 (50.0)
Coronary artery disease	0 (0)	0 (0)
Diabetes	0 (0)	0 (0)
COPD	0 (0)	1 (25.0)
Renal Failure	0 (0)	0 (0)

BMI, body mass index; COPD, chronic obstructive pulmonary disease; SD, standard deviation

Continuous variable are summarized by mean (standard deviation) and categorical variables are summarized by N (%).
