# Supplementary material for: Inhibition of vascular smooth muscle cell PERK/ATF4 ER stress signaling protects against abdominal aortic aneurysms
Source: JCI Insight. 2025 Jan 23;10(2):e183959. doi: 10.1172/jci.insight.183959 (PMC11790032; doi:10.1172/jci.insight.183959)

# Inhibition of Vascular Smooth Muscle Cell PERK/ATF4 ER Stress Signaling Protects Against Abdominal Aortic Aneurysms

**Authors:** Brennan Callow[1]; Xiaobing He[1]; Nicholas Juriga[1]; Kevin Mangum[1]; Amrita Joshi[1]; Xianying Xing[2]; Andrea Obi[1]; Abhijnan Chattopadhyay[3]; Dianna Milewicz[3]; Mary O'Riordan[4]; Johann E. Gudjonsson[2]; Katherine Gallagher[1,4]; Frank M. Davis.[1]

**Affiliations:** [1]Section of Vascular Surgery, Department of Surgery, University of Michigan. [2]Department of Dermatology, University of Michigan. [3]University of Texas Health Science Center at Houston. [4]Department Microbiology and Immunology, University of Michigan.

## Unedited blot and gel images

# Figure 1

**Figure 1D**

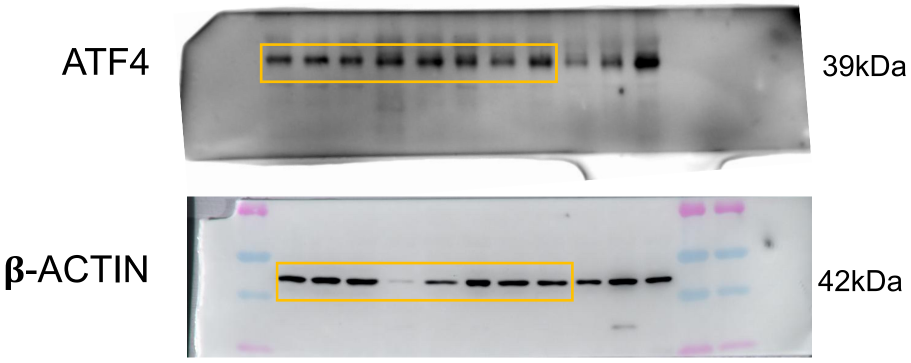


**Figure 1G**

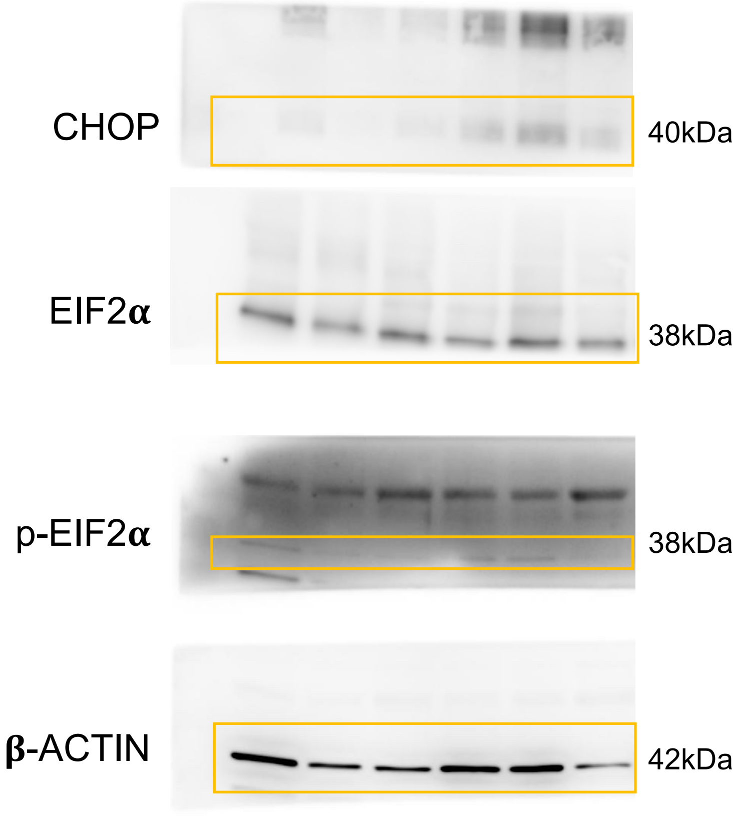


**Figure 1I**

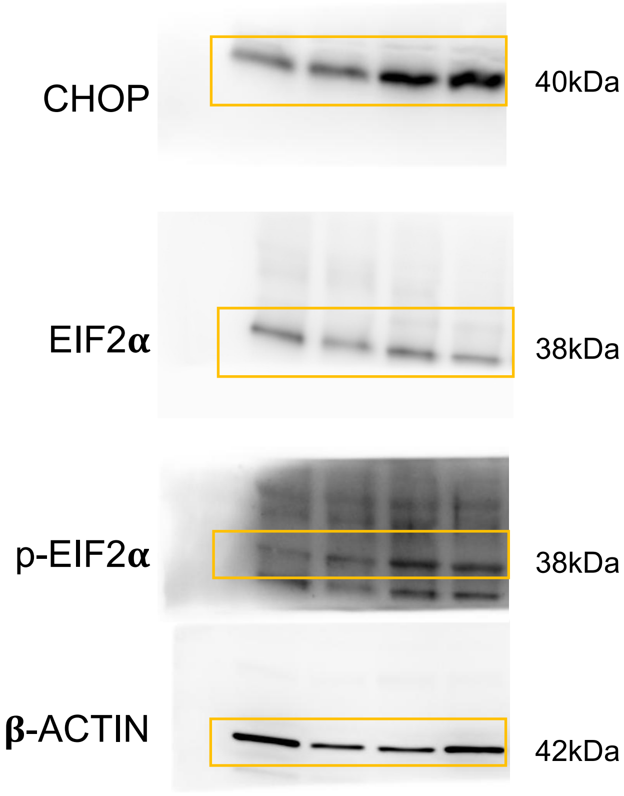

# Figure 2

**Figure 2B**

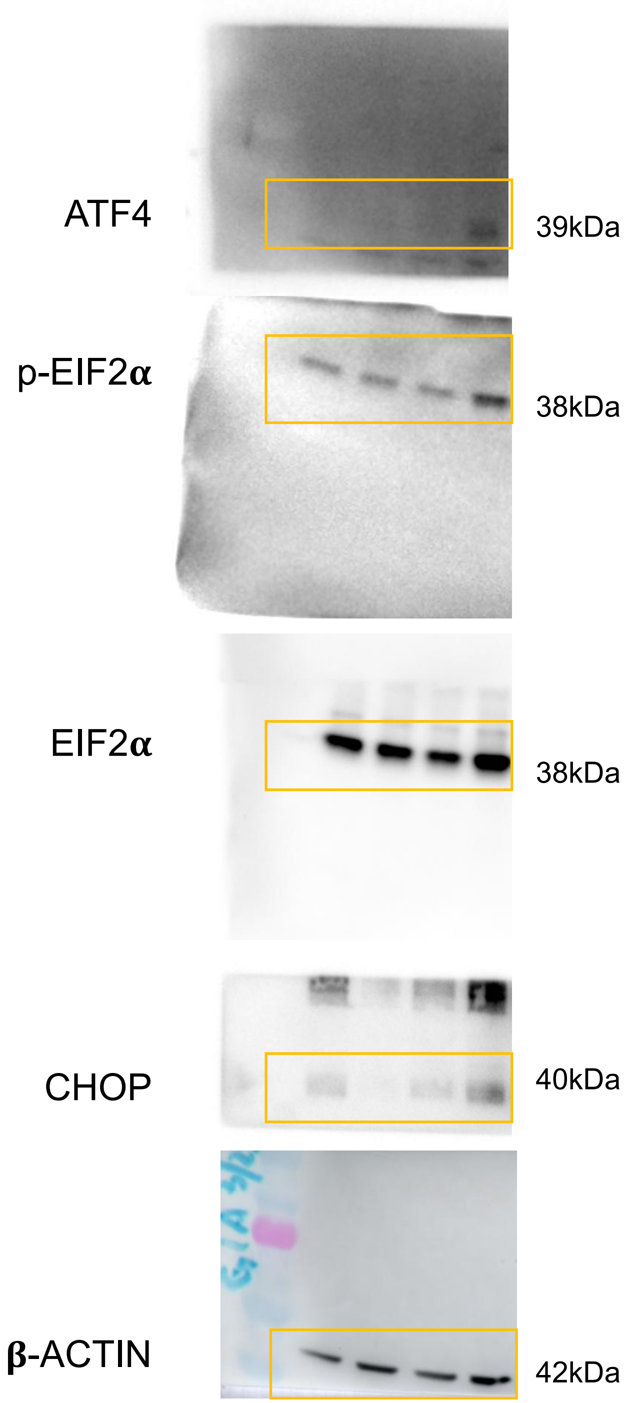

# Figure 4

**Figure 4B**

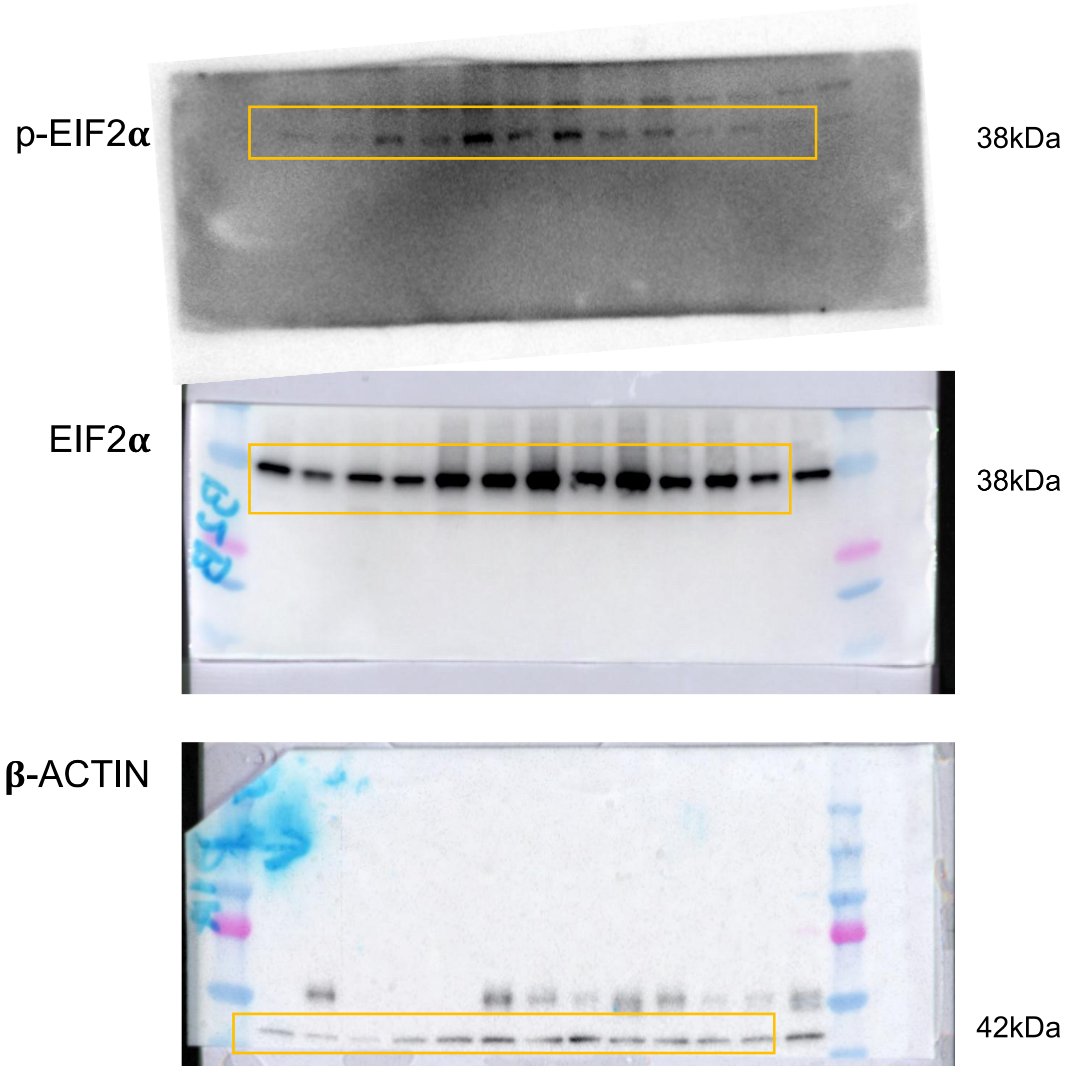

Supplement: Unedited blot and gel images [file jciinsight-10-183959-s190.pdf]
